# Sound Absorption Performance of Biobased Miura-Ori Origami Panel Absorbers Made from Impermeable Paper Membrane

**DOI:** 10.3390/polym18111287

**Published:** 2026-05-24

**Authors:** Luka Čurović, Anže Železnik, Andrej Hvastja, Jonas Trojer, Miha Brojan, Jurij Prezelj

**Affiliations:** Faculty of Mechanical Engineering, University of Ljubljana, Aškerčeva 6, 1000 Ljubljana, Slovenia; anze.zeleznik@fs.uni-lj.si (A.Ž.); andrej.hvastja@fs.uni-lj.si (A.H.); jonas.trojer@fs.uni-lj.si (J.T.); miha.brojan@fs.uni-lj.si (M.B.); jurij.prezelj@fs.uni-lj.si (J.P.)

**Keywords:** sound absorption, random incidence sound absorption coeffcient, biobased material, origami-based lightweight panel, Miura-ori structure

## Abstract

This study examines the potential of sustainable, biobased paper-based structures as panel/membrane sound absorbers. Although intact paper is naturally impermeable and a poor sound absorber, transforming it into complex three-dimensional origami geometries, specifically the Miura-ori pattern, could produce effective panel/membrane absorbers. Three distinct Miura-ori samples (A, B, and C) were fabricated with increasing geometric complexity, ranging from a simple triangular prism to a complex labyrinthine waveguide. The random incidence sound absorption coefficients of these samples were measured in a validated small-scale reverberation room. The underlying absorption mechanisms were further investigated through modal analysis and non-contact vibration velocity measurements. The results indicate that increased geometric complexity enhances acoustic performance. Sample C, the most complex structure, demonstrated the most consistent broadband absorption. The analysis confirmed a significant positive correlation between acoustic pressure modes, surface vibration velocity, and sound absorption peaks, indicating that acoustic energy dissipation is driven by the vibrational response of the paper membrane coupled with resonant modes in the air gap. This research demonstrates that tunable origami folding techniques using intact paper can be used to design lightweight acoustic treatments for diffuse sound fields in the mid-frequency range.

## 1. Introduction

Sound-absorbing materials are used in architecture and engineering for noise control and acoustic design. Commercially available sound absorbers are often made from non-renewable materials, such as mineral and glass fibres, polyurethane foams, and fibreglass [1,2,3,4]. In this context, sound-absorbing structures produced from biobased polymers offer a sustainable alternative [3].

Paper is a renewable material composed of a network of cellulose fibres extracted from natural resources. Its use in various applications provides significant environmental, technical, economic, and health benefits [4,5,6,7,8,9]. Research into paper and paper-based products for architectural and engineering applications has demonstrated that these materials can be used for sound absorption [5].

Paper absorbs acoustic energy through several mechanisms. When paper is returned to a loose state or to its primary component, cellulose, it acts as a porous sound absorber with cavities, channels, and interconnected pores where incident sound energy can be dissipated [5,9,10,11,12,13,14]. The sound absorption coefficient values of different porous paper structures depend on their specific microstructure, homogeneity, and thickness. Their frequency characteristics can be determined using empirical models if the airflow resistivity of the material sample is known [10,15,16].

Intact paper and cardboard have a dense structure and are considered impermeable; therefore, their sound absorption coefficient values are low [4,5,17]. Paper-based products can be transformed into effective absorbers with properties similar to porous samples by employing specialised geometry and orientation [15,16,18,19]. These absorbers attenuate sound through boundary layer friction and frictional interactions between paper layers of various shapes, or through dissipative losses as sound propagates through hollow “veins” or tubes, which should be arranged parallel to the direction of sound propagation. One acoustical effect of the tubes is that the sound wave impinging on the layer surface is forced to propagate inside the hollow layer in the direction normal to the surface [20]. Such absorbers require a thickness greater than 100 mm.

Another method to enhance the sound absorption properties of paper-based structures is by incorporating micro-perforations [5]. The acoustic performance of perforated cardboard panels has been examined in several studies [6,17,21]. These studies indicate that the sound absorption coefficient of perforated cardboard panels is comparable to that of conventional building materials at design frequency. Such structures achieve sound absorption through Helmholtz-type resonance, which depends on cavity depth, thickness, surface density, hole diameter, and perforation ratio [22,23].

When the perforation ratio is zero, the absorption mechanism shifts from the Helmholtz resonance MPP type to the panel or membrane type [23,24]. Recently, it was suggested that paper-based structures could be used as panel absorbers; however, a study on the sound absorption properties of paper-based panels has not yet been conducted [5].

The absorption characteristics of panel/membrane type absorbers should be studied in a reverberation chamber [6,25], as basic theoretical studies on panel or membrane absorbers were verified by comparing results with data obtained in a reverberation room [26,27]. However, most experimental studies on the sound absorption properties of paper structures have been conducted using the impedance tube method [4,6,10,12,13,14,15,16,17,18,19,28,29].

Paper is a thin, lightweight material. In practical applications, a single sheet of paper would not be used alone as a panel/membrane absorber; it would require support or clamping to ribs, which would affect the absorber’s frequency characteristics [25,26]. However, paper can be folded into complex three-dimensional shapes and is considered an ideal material for origami structures. Paper folds are flexible and sufficiently resistant to maintain their shape [19,30].

Folding paper into origami structures provides significant physical, mechanical, and functional advantages in various engineering and architectural acoustics applications. Origami and paper folding techniques have been used to enhance the absorption properties of quarter-wavelength resonators [30], and in the design of tunable Helmholtz resonators [31], duct silencers [32], micro-perforated resonators [33], reconfigurable silencing windows [34], foldable and portable sound barriers [35], structures with tunable waveguiding features [36], sound barriers based on structures with tunable waveguiding features [37], and acoustic waveguides [38].

However, none of the studies considered the use of an origami paper structure as a panel/membrane absorber in a diffuse sound field, where it is expected that performance could improve, as the uneven surfaces of the origami structure follow the incidence angle of a random sound field [2,27,39,40,41].

The effect of the absorber’s surface depends on the underlying sound absorption mechanism and the type of material used. Recently, cylindrical sound absorbers with flat and origami-shaped surfaces were studied in a large reverberation room. The folding pattern was selected to achieve optimal luminescence distribution and design flexibility, while the surfaces consisted of a permeable membrane. The absorber was used as a space absorber without an air cavity, not as a panel absorber. The results showed no significant difference between absorbers with flat and origami surfaces [2]. On the other hand, the use of metal wavy structures and sinusoidal surfaces with a closed cavity behind has shown that the sound absorption properties of micro-perforated panels are improved [40].

According to published research, cellulose has previously been studied as a porous sound absorber, while paper-based sound-absorbing panels have been analysed as micro-perforated panels. To our knowledge, a study on the sound absorption properties of intact, impermeable paper-based panel/membrane sound absorbers has not been conducted. Research on such composites may be hindered by difficulties in scaling up, their light weight, anisotropy and a lack of studies conducted in diffuse field situations [42,43].

To address this gap, the study considers Miura-ori origami folded paper-based structures as panel/membrane absorbers. Origami panel absorbers are expected to have an effect in diffuse sound fields, where uneven surfaces encounter various incidence angles. The geometrical features of the panels are presented, and it is shown how systematically increasing the geometric complexity of the Miura-ori pattern affects acoustic performance and broadband sound absorption, measured in a validated small reverberation room. The underlying sound absorption mechanisms are investigated through numerical modal analysis and vibration velocity measurements. The sound absorption performance of paper-based panels is compared with that of a membrane absorber made from nonwoven polymer material.

## 2. Materials and Methods

### 2.1. Material Characterization

#### 2.1.1. Specimen Preparation

**Figure 1:**
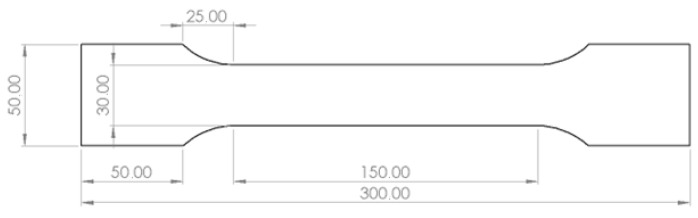
A sketch of the specimen and its geometry.

The mechanical properties of the paper material used to fabricate the origami samples were experimentally determined through uniaxial tensile testing. All specimens were laser cut from the same paper sheet to minimise variability due to material heterogeneity. Owing to the anisotropic nature of paper, specimens were prepared along both the machine direction (*M*) and the cross direction (*C*), with three specimens tested in each direction. Custom specimen geometry was used for the tensile tests, as shown in Figure 1.

Prior to testing, all specimens were conditioned for 24 h at a temperature of 23 °C and a relative humidity of 30% to reduce the influence of environmental conditions on the measured mechanical response.

#### 2.1.2. Mechanical Characterization of the Specimen

**Figure 2:**
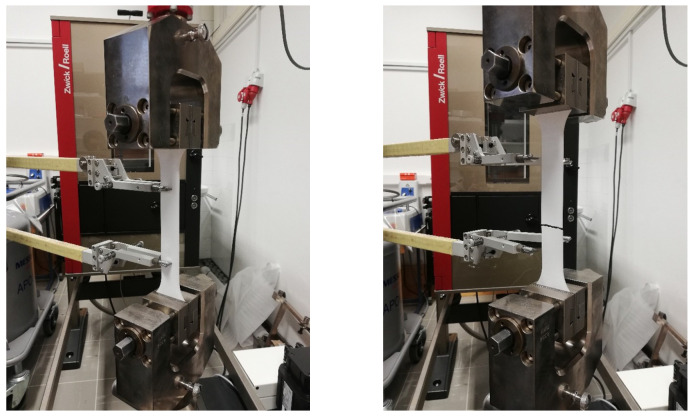
A specimen before (**left**) and after the test (**right**).

Uniaxial tensile tests were conducted using a Zwick/Roell Z100 universal testing machine fitted with a Zwick/Roell 5 kN HP+ load cell and a MultiXtens extensometer. The experimental setup is shown in Figure 2.

The tests were conducted under displacement-controlled conditions without preload. During the initial linear elastic region used to determine Young’s modulus, the crosshead speed was set to 1 mm/min. After completing the modulus evaluation range, the loading speed was increased to 50 mm/min until specimen failure. The displacement sampling resolution was 1 μm.

Engineering stress was calculated as $\sigma =F/A_{0}$ where *F* is the measured tensile force and $A_{0}$ is the initial cross-sectional area of the specimen. Engineering strain was calculated as $\epsilon =\Delta L/L_{0}$ where ΔL is the measured elongation and $L_{0}=100$ mm is the initial gauge length.

Young’s modulus was determined from the stress–strain response using linear regression within the strain interval of 0.05% to 0.25%. This range was chosen to capture the approximately linear elastic region while minimising the influence of initial seating effects and nonlinear deformation at higher strain.

**Figure 3:**
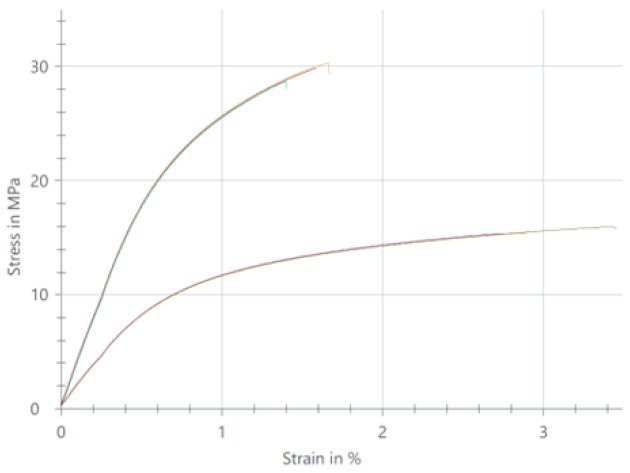
Stress–strain curves for tested specimens. A clear difference between the machine and cross direction specimens is recognized.

The measured engineering stress–strain curves for all tested specimens are shown in Figure 3. A clear difference in stiffness between the machine and cross directions is evident.

The obtained Young’s modulus values in the machine ($E_{M}$) and cross directions ($E_{C}$) are $E_{M}=\left(3736.69\pm 12.64\right)$ MPa and $E_{C}=\left(1712.27\pm 6.16\right)$ MPa.

**Figure 4:**
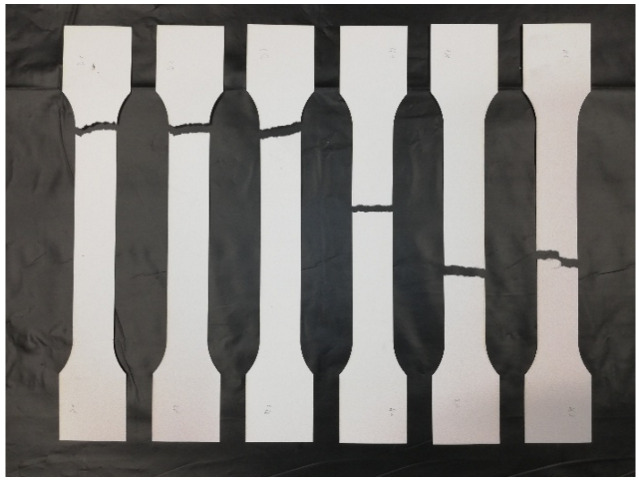
Specimens after the test.

All specimens exhibited snap failure. For specimens oriented in the machine direction, fracture typically occurred outside the extensometer gauge region, whereas those oriented in the cross direction fractured within the gauge section, as shown in Figure 4. No delamination or fibre pullout was observed during testing. Representative fracture photographs are shown in Figure 4.

### 2.2. Geometry and Fabrication of the Samples

**Figure 5:**
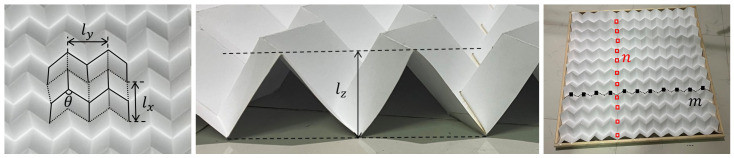
Geometry of the Miura cells ($l_{x},l_{y},l_{z},\theta$) and origami sample (m,n).

The origami samples were made from paper using the Miura-ori pattern. The density of the paper was 758.5 kg/m^3^, and its thickness was 0.244 mm. A Miura structure can be described by six parameters [44]. These parameters are the height ($l_{z}$), length ($l_{x}$), and width ($l_{y}$) of the cell, the number of cells in the *x* (*m*) and *y* (*n*) directions, and the sector angle (θ) [44]. The basic geometry of the Miura cell is shown in Figure 5.

All structures used in this study had the same cross-section: an equilateral triangle with $l_{x}=72$ mm, $l_{z}=45$ mm, and cell width $l_{y}=72$ mm, while the sector angle varied from 180° for sample A, 113° for sample B, to 55° for sample C. The height of the cells ($l_{z}$) was considered the standard height used in room acoustics applications in the mid-to-high frequency range. $l_{x}$ and $l_{y}$ were chosen arbitrarily; however, 72 mm wide cells can be considered typical for paper models, are easy to manipulate, and can hold their shape.

Thus, the shape of their internal volume changed from contiguous and unobstructed (sample A) to labyrinthine (samples B and C).

Sample A is a straight triangular prism. Its boundaries are parallel to the axis of wave propagation, and the volume is contiguous and unobstructed. Sample B introduces periodic perturbations, increasing the complexity of the structure in terms of surface area and acoustic path. Sample C is a complex labyrinth waveguide, consisting of a series of discrete units in a “zig-zag” pattern. The transition between these structures represents a systematic increase in geometric complexity, surface area, and acoustic tortuosity.

**Figure 6:**
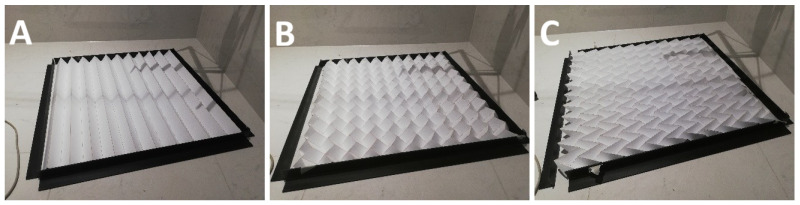
Origami samples (**A**–**C**).

The overall dimensions of the samples tested in the alpha chamber were limited to 800 × 800 mm. Accordingly, the two remaining parameters, *m* and *n*, required to fully define the Miura structure (i.e., the number of cells in the $l_{x}$ and $l_{y}$ directions), were determined to satisfy the overall dimension constraint. The tested parameter combinations for the three samples are listed in Table 1. Geometric tortuosity ($\tau_{g}$), calculated as the ratio of the length of the tortuous path to the straight-line length in the *y* direction [45], and the surface area of the sample (*A*), which are used to characterise the complexity of the origami panels, are also presented in Table 1. The origami samples are shown in Figure 6.

**Figure 7:**
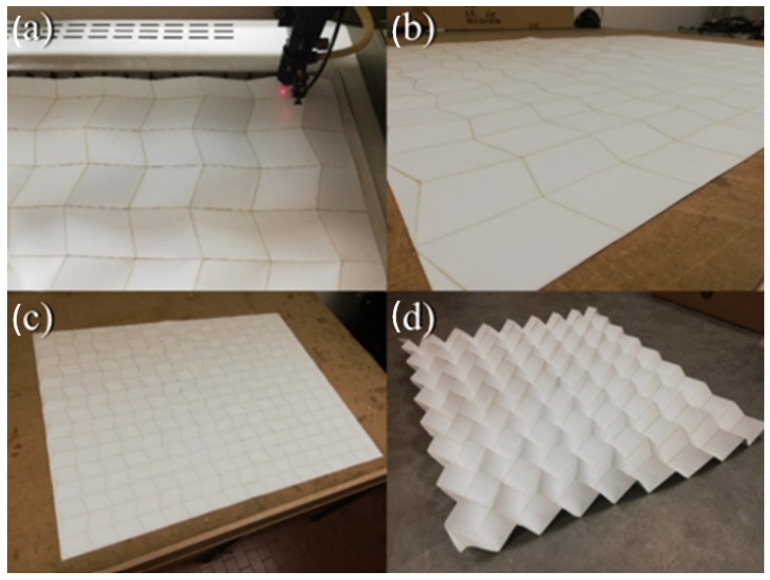
Fabrication of the Miura samples. Laser engraving (**a**), engraved pattern in the flat configuration (**b**,**c**), and final folded configuration (**d**).

The samples were produced by engraving the desired Miura patterns onto paper using a CNC laser cutter, then folding the paper by hand along the engraved lines, as shown in Figure 7.

### 2.3. Sound Absorption Coefficient Measurements in a Small Reverberation Room

#### 2.3.1. Theoretical Model

The (random incidence) sound absorption coefficient of a panel can be evaluated using the theoretical characteristic of a plate backed by a cavity of non-uniform depth in a diffuse sound field [26,27]. The input parameters of the complex theoretical model include the thickness of the paper sheet (*h* = 0.244 mm), paper density (ρ = 758.4 kg/m^3^), Young’s modulus (*E* = 1712 MPa and 3737 MPa), loss factor (η = 0.0001), Poisson’s ratio (σ = 0.3), and admittance of the back wall ($A_{b}$ = 0.005). The depth of the air back cavity was chosen as the average cavity depth, 22.5 mm [27]. The angle of incidence was varied between 15° and 60°, and the envelope of the absorption peaks is presented as the final result.

#### 2.3.2. Measurements

**Figure 8:**
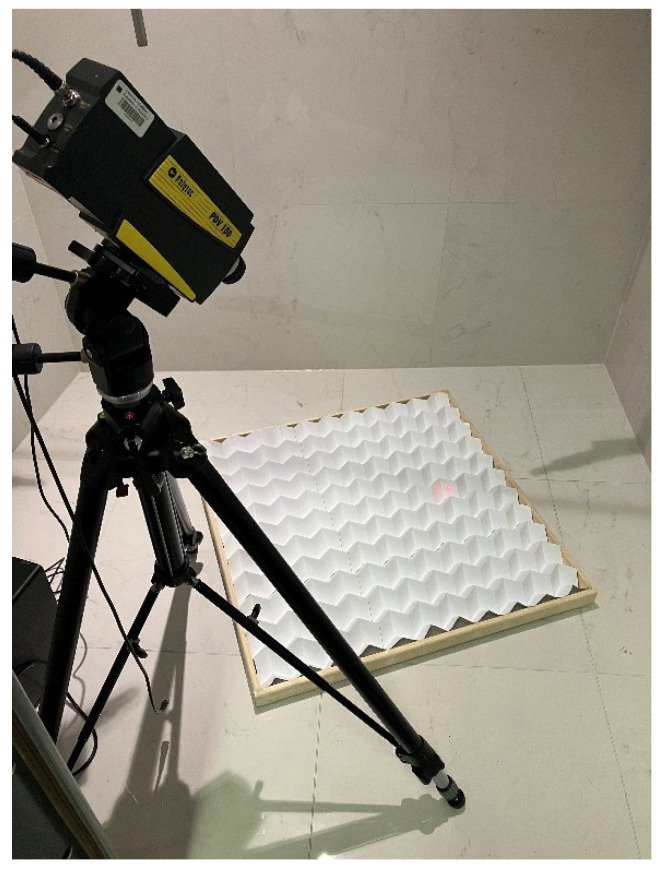
The non-contact vibration velocity measurements performed with a 1D laser Doppler vibrometer Polytec PDV100.

The measurements were conducted in a small reverberation room with six non-parallel surfaces, a volume of 6.4 m^3^, and an approximate surface area of 21 m^2^. The room is shown in Figure 8. The walls and ceiling comprised two layers of plaster, and the floor was a thick concrete slab. All room surfaces, including doors, were covered with ceramic tiles. The Schroeder frequency of the room was estimated to be above 900 Hz. To minimise large variations in the low frequency range, the number of measurements was increased to forty, including eight different microphone positions and five different loudspeaker positions. At each microphone and loudspeaker position, four decay curves were obtained in each frequency band of interest [46]. The room was validated using materials with known sound absorption coefficient values [43,47]. Two mineral wool samples, with thicknesses of 50 mm and 100 mm and an approximate density of 40 kg/m^3^, were selected for this purpose. The absorption coefficient values measured in a small room were consistent with the data obtained in a standard reverberation room, according to the manufacturer’s specifications. Absorption measurements were conducted using a noise-interrupted noise method, following guidance proposed by SAE [48]. Pink noise served as the excitation signal. The signal duration was set at 9 s, and recording continued for an additional 9 s after the excitation was interrupted.

Generation of the pink noise excitation signal, microphone signal acquisition, and digital signal processing (filtering and least-square fitting) were carried out using custom NI LabView software. Excitation signals were sent to a sound card (Motu StageB16) and played through a loudspeaker (Yamaha HS 5 with 5-inch cone woofer and 1-inch dome tweeter, 54 Hz–30 kHz frequency response). Signals were recorded at a sampling frequency of 96,000 Hz using eight half-inch microphones (BK type 4145, sensitivity 50 mV/Pa, dynamic range 10.2 to 146 dB, frequency range 2.6 Hz to 18 kHz), an amplifier (BK type 2636), and a sound card (Motu StageB16).

The tested specimens were placed directly on the room floor. Each sample was placed in an oblique position in which none of its edges were parallel to the walls or ceiling. The edges were covered with a sound-reflecting frame made of 12.5 mm wood. Sound absorption was measured four times for each sample. Between each repetition, the sample was disassembled, repositioned on the floor, and put in the frame.

#### 2.3.3. Evaluation of Performance

The acoustic performance was evaluated using the peak value of the sound absorption characteristic curve ($SAC_{peak}$) and the average sound absorption coefficient (ASAC) in the frequency range from 125 Hz to 4000 Hz [49].

### 2.4. Acoustic Modes in the Air Gap—Numerical Model

Using numerical modelling and modal analysis, it was shown that acoustic modes in the air gap (backing cavity) significantly affect the sound absorption characteristics of wavy and corrugated microperforated panels [40,41,50]. In this study, the complex geometry of origami membrane/panel absorbers was investigated by considering the acoustic modes of the air cavity between the origami panel and the reflecting floor. The resonant eigenfrequencies ($f_{m}$) of the air cavity were numerically calculated using the finite element method (FEM) implemented in COMSOL Multiphysics version 6.4. As the origami was placed on the room floor, the analysis focused on a single line of the original origami element. The boundaries of the cavity were defined as “acoustically rigid walls” [41,50]. A predefined physics-controlled mesh in COMSOL with a very fine element size was used, and the air temperature was set at 293.15 K. For each configuration, the first forty resonant frequencies and their corresponding eigenmodes (eigenvalues) were calculated.

### 2.5. Vibration Velocity Measurements

Vibrations and structural response of finite membrane/panels are an important mechanism for sound energy dissipation [25,51,52,53,54]. Paper is a thin, lightweight material where mass loading from vibration sensors should be avoided [55,56,57]. In the experiment, the room and the origami pattern were excited using a loudspeaker and a pink noise signal. Non-contact vibration velocity measurements on the panel surface were performed with a Polytec PDV100 1D LDV [57,58]. The vibrometer was mounted on a tripod, as shown in Figure 8. Paper exhibits anisotropic dynamic properties, and to capture both out-of-plane and in-plane structural dynamics, angled positioning of the laser vibrometer was used. Data acquisition and signal processing were carried out using Measurement Computing acquisition hardware (MC 1608FS+) and NI LabView software. The sampling rate was 50 kHz. An amplitude measurement range of 20 mms^−1^ with a sensitivity of 25 mms^−1^V^−1^ was used. A Hanning window of size 212 with 80% overlap was used to calculate the one-sided power spectral density (PSD). The signal was sampled for 30 s to obtain an appropriate signal-to-noise ratio.

### 2.6. Structural Modal Analysis Using Numerical Methods

#### 2.6.1. Finite Element Model

The modal analysis of the investigated Miura-ori structures was conducted using ANSYS Mechanical APDL 2022 R2. The folded geometry was analytically generated based on the idealised Miura fold pattern, using parameters from Table 1. The numerical model assumed perfectly sharp fold lines, without geometric imperfections or local fold radii. Fold lines were modelled without local stiffness reduction or hinge weakening. The structure was discretised using SHELL181 quadrilateral shell elements. The simulations assumed linear elastic material behaviour and small structural deformations. The mesh consisted exclusively of quadrilateral elements with near-unity aspect ratios to minimise numerical distortion effects and ensure uniform discretisation quality across the folded geometry. For all analyses, each individual parallelogram facet of the Miura pattern was discretised. Mesh convergence was assessed by comparing a selected eigenshape of an individual facet with a natural frequency of approximately 200 Hz, using mesh sizes of 5 × 5, 8 × 8, and 10 × 10 parallelograms. Relative differences in the calculated natural frequencies below 1% were considered acceptable.

#### 2.6.2. Material Model

An orthotropic linear elastic material model was used to represent the anisotropic mechanical behaviour of the paper. The local material *x*-direction was aligned with the machine direction (*M*) of the paper, while the local *y*-direction corresponded to the cross direction (*C*). Only the in-plane Young’s moduli in the principal material directions were experimentally determined through tensile testing. The values used in the numerical model were $E_{x}=3736.7$ MPa and $E_{y}=1712.3$ MPa. A Poisson ratio of $\nu_{xy}=0.3$ was selected based on a sensitivity analysis that investigated the influence of varying Poisson ratio values on the calculated natural frequencies. The remaining orthotropic constants required by the ANSYS material model were determined using the following relations. The reciprocal Poisson ratio was calculated as:

(1) $$\nu_{yx}=\nu_{xy}\frac{E_{y}}{E_{x}}.$$

The in-plane shear modulus was estimated using the following relation:

(2) $$G_{xy}=\frac{E_{x}}{2(1+\nu_{xy})}.$$

The average measured thickness of t=0.244 mm was assigned uniformly to all shell elements in the numerical model. The calculated density of ρ=758.5 kg/m^3^ was assigned uniformly throughout the finite element model.

#### 2.6.3. Boundary Conditions and Modal Extraction

Boundary conditions were defined by constraining the out-of-plane displacement component ($u_{z}$) at all nodes in contact with the supporting surface. This approach approximated the simple support conditions of the plate resting on the floor of the reverberation room, while allowing in-plane deformation and rotational freedom. The natural frequencies and corresponding mode shapes were extracted using the Block Lanczos eigensolver implemented in ANSYS MAPDL 2022 R2. The first 4000 eigenfrequencies were calculated to capture the complete modal density within the investigated acoustic frequency range.

## 3. Results and Discussion

### 3.1. Sound Absorption Coefficient

**Figure 9:**
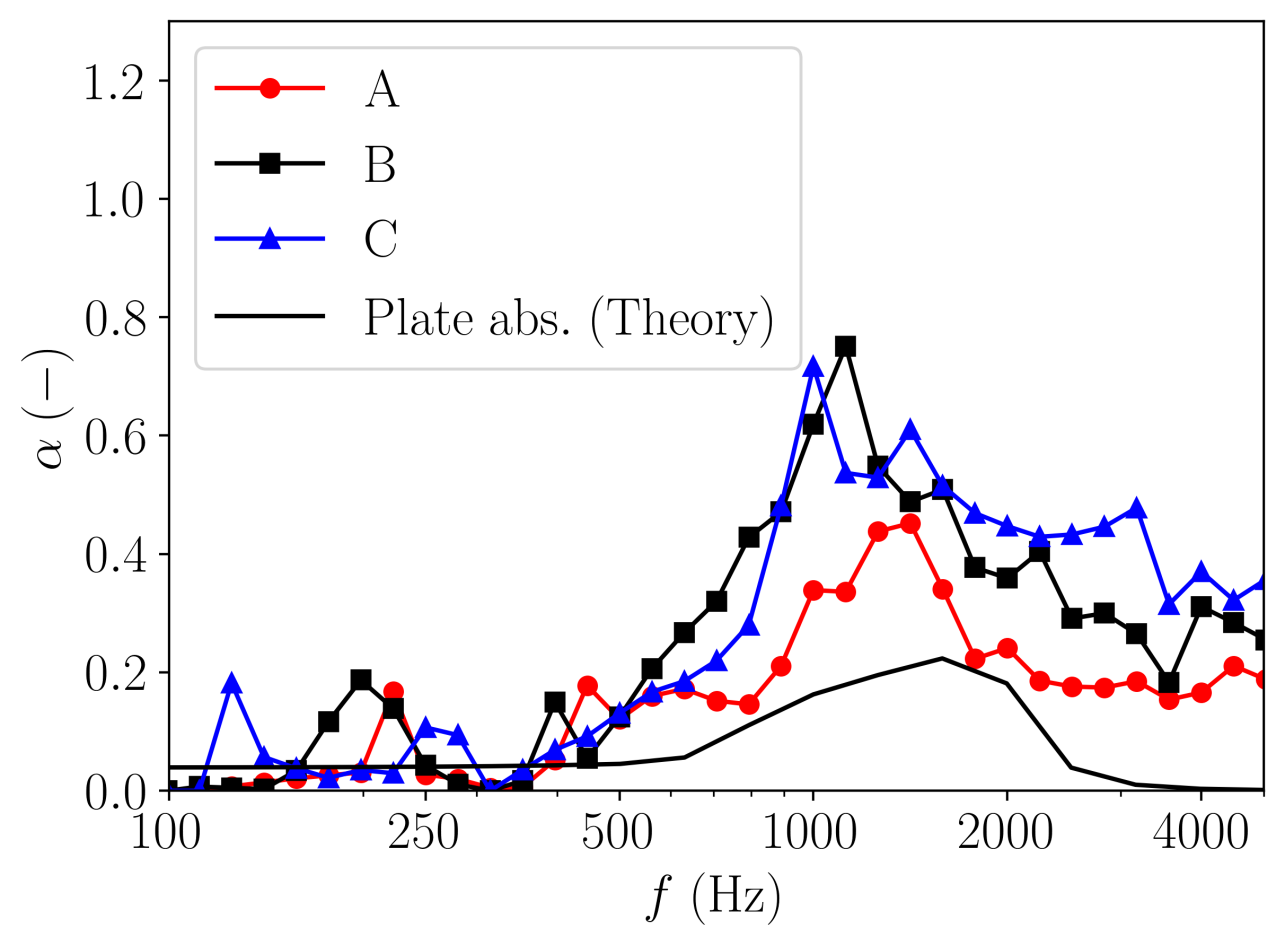
Sound absorption coefficient of origami samples and theoretical characteristic of a plate backed by a cavity of non-uniform depth.

The (random incidence) sound absorption coefficient of the three origami structures and the theoretical characteristic of a plate backed by a cavity of non-uniform depth in a diffuse sound field [26,27] are shown in Figure 9.

The most prominent feature of the theoretical curve is a significant absorption peak, caused by the resonant vibration of the system (the plate’s mass coupled with the air cavity’s stiffness), which is also observed in the measured data for the three samples.

According to the theoretical model, it is expected that once the resonant frequency is exceeded, the absorption coefficient of a plate absorber decreases.

However, paper-based origami structures are lightweight and can maintain a coefficient of 0.30–0.50 even at higher frequencies, which is characteristic of porous materials. All sound absorption curves of origami samples display a series of spectral peaks where the absorption coefficient reaches a local maximum, as well as a more continuous broadband absorption pattern beginning at around 500 Hz. The clarity of the peaks indicates that sound absorption maxima are achieved through resonance effect.

Different geometries result in different frequency characteristics of the sound absorber. Sample A has small low-frequency peaks at around 250 Hz and 445 Hz, then shows a gradual increase in absorption starting at around 500 Hz, peaking at $SAC_{peak}=$ 0.45 in the 1260 Hz to 1414 Hz frequency range. After this peak, performance drops and remains around 0.15–0.20 in the high-frequency range (>2500 Hz). The average sound absorption coefficient (ASAC) of the sample A in the frequency range 125 Hz to 4000 Hz is 0.16.

The low-frequency peaks of sample B are at 198 Hz and 397 Hz. It reaches a strong peak of 0.75 at 1122 Hz and maintains high absorption between 800 Hz and 1500 Hz. The performance of sample B decreases at very high frequencies but remains above 0.20. ASAC of the sample B is 0.25.

Sample C has a flatter response compared to samples A and B. The frequency peaks occur around 125 Hz and between 250 Hz and 281 Hz. Although its main peak is slightly lower (0.72 at 1000 Hz), it maintains a more consistent absorption level (around 0.30–0.50) across the higher frequencies (2000 Hz to 3000 Hz). ASAC of the sample C is 0.27. Based on the value of ASAC sample C is the most effective absorber overall, followed by Sample B and then Sample A.

### 3.2. Acoustic Modes

#### 3.2.1. Resonant Frequencies

**Figure 10:**
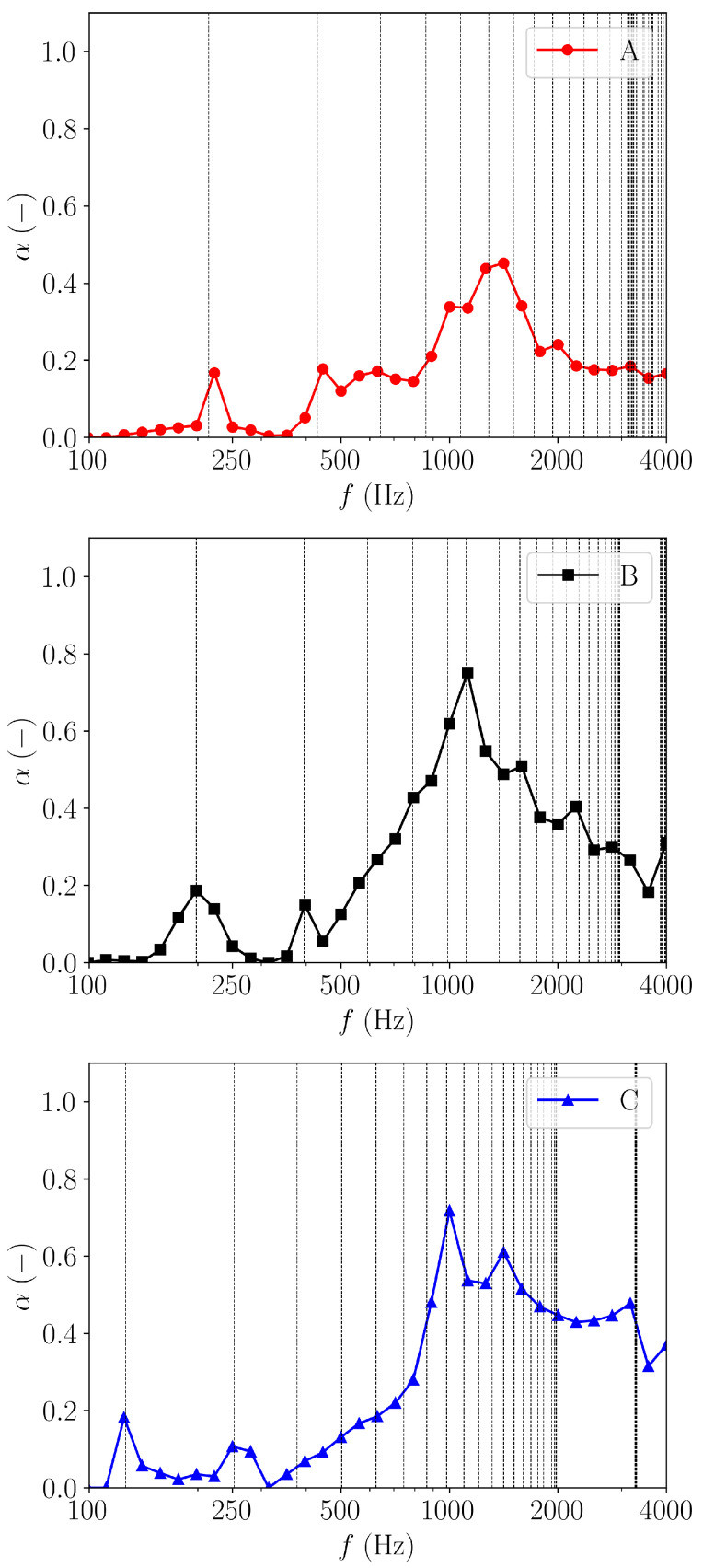
Sound absorption coefficient and resonance frequencies (vertical lines) of the air gap for samples A, B and C.

The acoustic eigenfrequencies of the cavity for the three samples are shown in Figure 10. Their positions are indicated by vertical lines, which are displayed alongside sound absorption coefficient data. The three samples exhibit distinct behaviours in the distribution of their eigenfrequencies across the spectrum. Sample A has the highest frequency values for the corresponding modes. The spacing between the frequencies is relatively consistent, with at least two modes in every 500 Hz bin. The modal density increases above 3000 Hz. Samples B and C both exhibit clear gaps where the modal density drops to zero. For sample B, there is a notable gap and absence of resonant modes between 2965 Hz and 3847 Hz. Sample C has the lowest first eigenfrequency (126 Hz), no modes between 2000 Hz and 3270 Hz, and a high modal density between 3272 Hz and 3300 Hz.

By examining eigenfrequency and absorption data together, we can see that cavity resonances significantly affect sound absorption performance.

For sample A, the highest absorption occurs in the 1259.9 Hz and 1414.2 Hz 1/6 octave frequency bands, which are correlated with the sixth and seventh cavity modes at discrete frequencies of 1284 Hz and 1502 Hz.

The performance of sample B is also linked to the identified acoustic eigenfrequencies of the cavity. The positions of the absorption peaks correspond to the first, second, and sixth modes. The frequency range of increased absorption compared to sample A includes the eigenfrequencies of the fourth to tenth modes. The performance decreases in the 3000 Hz to 4000 Hz range, where there are no modes.

The absorption peaks at 1000 Hz and 1414 Hz for sample C are associated with the 8th and 11th cavity modes, while the absorption peak at 3175 Hz occurs where there is a high modal density around 3300 Hz. The dips in the 2000 Hz to 3000 Hz range and at 3563 Hz may be explained by larger gaps in the eigenfrequency distribution.

#### 3.2.2. Acoustic Pressure

**Figure 11:**
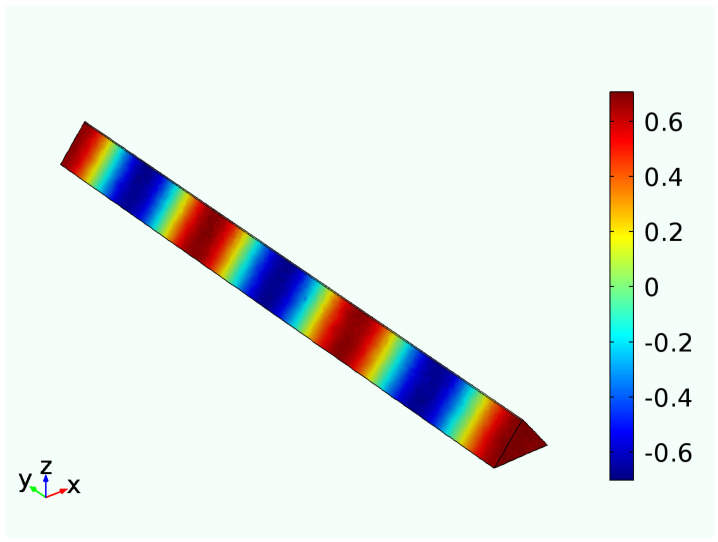
Sample A: acoustic mode at f(m=6)= 1287 Hz.

Figure 11 shows the acoustic pressure distribution (mode shape) of the sixth mode at 1287 Hz in the air gap defined by the paper surface of the origami element and the floor of the room for sample A. It displays a longitudinal standing wave along the length of the sample. The wave fronts are relatively uniform and perpendicular to the axis of the cavity.

**Figure 12:**
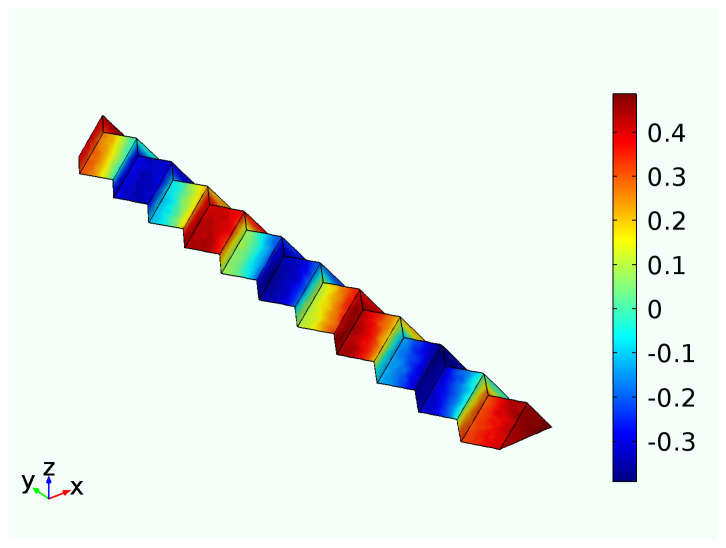
Sample B: acoustic mode at f(m=6)= 1179 Hz.

Figure 12 shows the acoustic pressure distribution (mode shape) of the sixth mode at 1179 Hz in the air cavity of sample B. The eigenfrequency of the mode decreases as additional geometrical features effectively increase the acoustic path length. The wave fronts are no longer perfectly flat. The geometric discontinuities scatter the sound field slightly, resulting in a less uniform pressure distribution compared to that in sample A. The pressure distribution remains continuous. Although the boundaries of the cavity are staggered, the acoustic energy flows smoothly from one peak to the next. The nodes (the green and yellow zero-pressure zones) are relatively wide, indicating a gradual transition typical of a standard standing wave.

**Figure 13:**
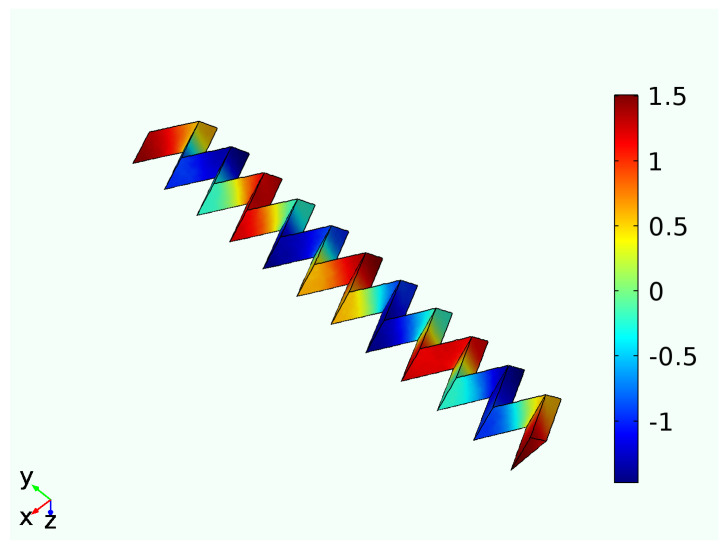
Sample C: acoustic mode at f(m=8)=982 Hz.

The acoustic pressure distribution of the eighth mode at 982 Hz in the air cavity of sample C is shown in Figure 13. As the geometry becomes more complex and discontinuous, the mode resembles less a single cohesive longitudinal wave and more a series of coupled local resonances. The pressure maxima (dark red) and minima (dark blue) are concentrated within the individual segments. The pressure transition between segments is much sharper. The structure begins to behave like a chain of individual resonators. The pressure is highly localised within the diamond-shaped segments. The transition between positive and negative pressure zones occurs very abruptly at the narrow junctions between segments, which is characteristic of a high-tortuosity path. This feature enhances sound attenuation and improves sound absorption due to boundary layer friction [15,16,19].

### 3.3. Vibrational Velocity

**Figure 14:**
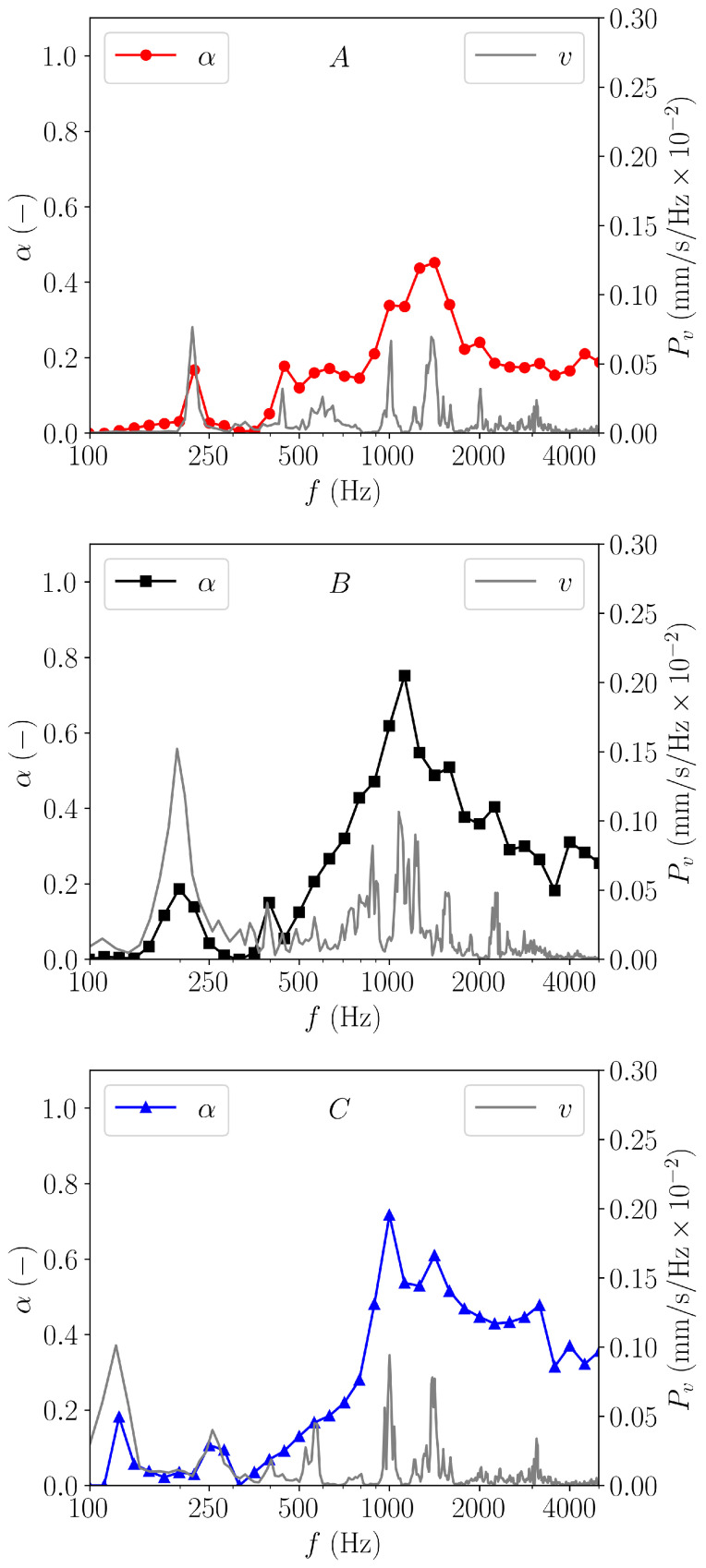
Sound absorption coefficient and power density spectra of vibration velocity on the surface of origami panels (**A**–**C**).

The vibrational velocity spectra and sound absorption coefficient data for the three samples are shown in Figure 14. There is a significant positive correlation between these two physical properties across the frequency spectrum. Strong vibrations of the origami surface are observed at the corresponding absorption peaks. The data suggest that dissipation of acoustic energy due to vibration of the paper structure, induced by cavity modes, is an important mechanism for sound absorption.

### 3.4. Structural Modal Analysis

**Figure 15:**
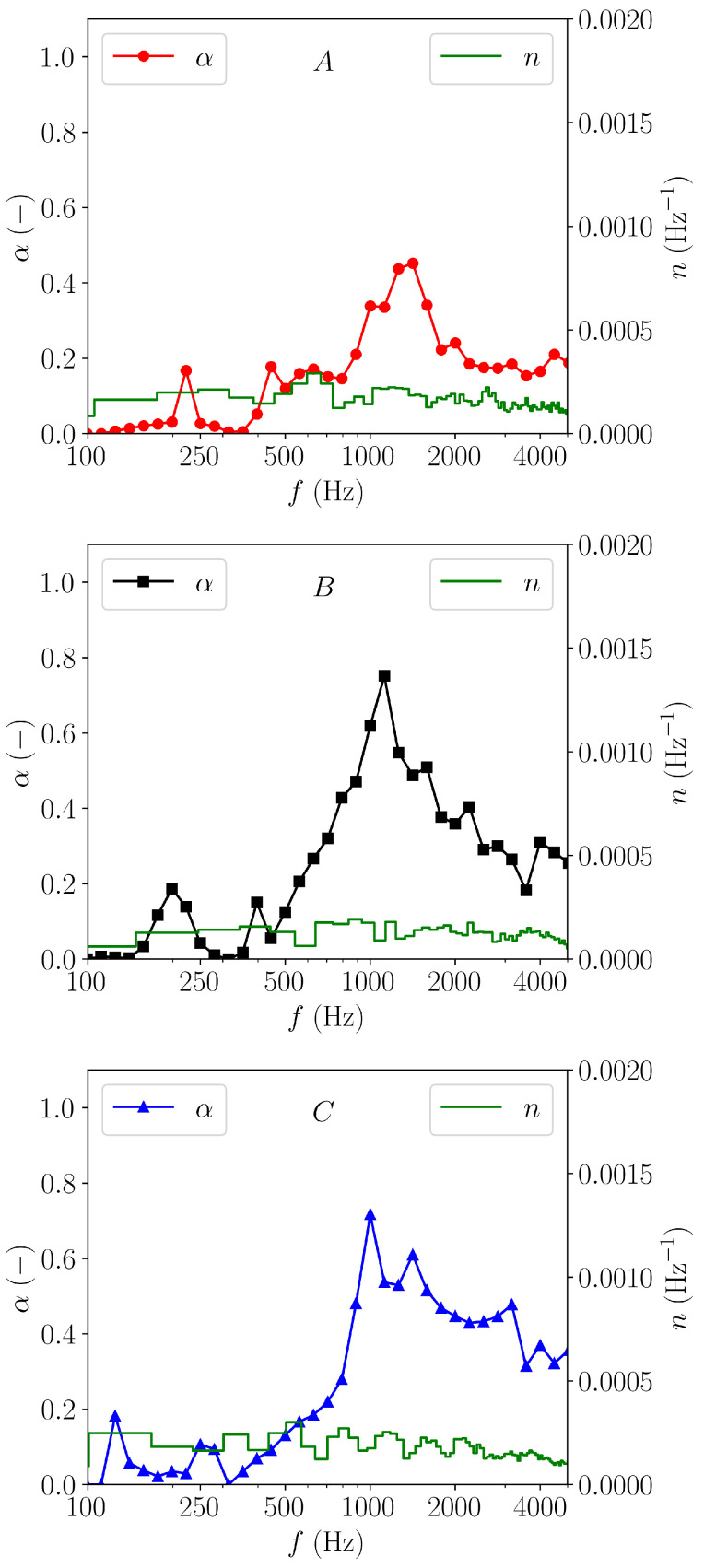
Sound absorption coefficient and density distribution of eigenfrequencies for origami paper-based panels (**A**–**C**).

The density distribution of the first 4000 eigenfrequencies, obtained using the Block Lanczos numerical model and sound absorption data for the three samples, is shown in Figure 15. The distributions above 100 Hz are uniform, and there is no clear correlation between modal density and the positions of the sound absorption peaks.

### 3.5. Comparison with Other Materials

#### 3.5.1. Comparison with Commercially Available Material

**Figure 16:**
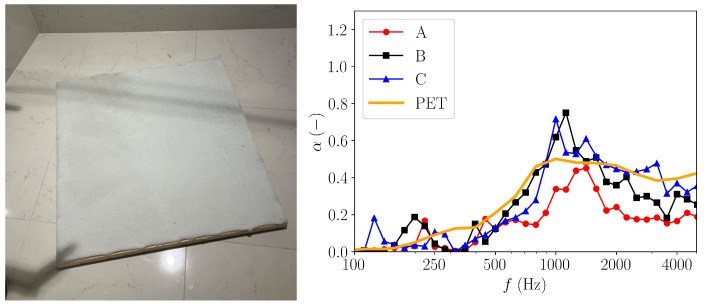
Membrane PET-based panel (**left**) and sound absorption coefficient data (**right**).

The performance of the origami samples was compared with a membrane panel made from commercially available technical nonwoven textile material composed of polyethylene terephthalate (PET) fibre. The measured flow resistivity of the membrane was 4075 Pa.s/m^2^ and its surface density was 0.4 kg/m^2^. The membrane was placed on the same wooden frame used to cover the edges of the origami samples. Therefore, the air gap behind the membrane was 4.5 cm, which was equal to the height ($l_{z}$) of the origami samples. The sound absorption coefficient characteristic of the PET-based membrane panel and the three origami samples is shown in Figure 16. The ASAC of the PET-based panel was 0.26, and $SAC_{peak}$ was 0.5 at 1000 Hz. Both values were below those of origami sample C.

#### 3.5.2. Comparison with Previous Research Results

Recently, transparent wood biocomposites used as membrane panels for indoor environments were studied [59]. Laboratory tests using an impedance tube showed that the highest absorption occurred at 630 Hz ($SAC_{peak}$ = 0.659 for maple wood and 0.603 for mahogany wood), with absorption rates exceeding 20% at frequencies ranging from 400 to 3150 Hz. ASAC values were not presented but can be estimated at approximately 0.25.

Absorbers made from High-Pressure Laminate (HPL) designed for low-frequency sound were tested in a reverberation room [60]. The thinner 1.3 mm HPL panels exhibited relevant sound absorption peaks below 200 Hz, while sound absorption coefficients above 200 Hz remained below 0.2. The ASAC of these panels is low, as expected, since they were specifically designed for low-frequency sound absorption below 200 Hz, where paper-based composites are less effective.

The ASAC and $SAC_{peak}$ values of origami samples B and C were higher than those of transparent wood biocomposites and absorbers made from High-Pressure Laminate (HPL) in the mid-frequency range.

## 4. Conclusions

This study investigated the potential of biobased, paper-based origami structures as sustainable alternatives to non-renewable sound-absorbing materials. Although intact paper is naturally impermeable and usually a poor acoustic absorber, converting it into three-dimensional origami structures produces efficient panel/membrane absorbers. The research demonstrates that systematically increasing geometric complexity—from a simple prism (Sample A) to a complex labyrinthine waveguide (Sample C)—significantly enhances acoustic performance.

The investigation into the underlying mechanisms reveals that sound absorption is enhanced by resonant acoustic pressure modes within the backing air cavity, which are coupled with the vibrational response of the lightweight, anisotropic paper membrane. Numerical modal analysis and vibration measurements showed a significant positive correlation between these factors and the measured absorption peaks. Specifically, Sample C, the most complex structure, exhibited several low-frequency peaks, a high absorption peak at 1000 Hz, and the most consistent broadband absorption. Furthermore, the study highlights that increased acoustic tortuosity in complex designs such as Sample C promotes sound attenuation through localised resonances and boundary layer friction within individual segments of the structure.

These findings indicate that tunable origami folding techniques offer a practical approach for designing lightweight, renewable acoustic treatments for diffuse sound fields in the mid-frequency range. Such structures may be relevant for improving speech intelligibility and reducing noise in offices or classrooms where mid-frequency noise is dominant.

Future research may focus on optimising the parameters of the Miura-ori cell for specific frequency ranges and assessing their durability in practical, large-scale applications, which can be achieved by assembling several smaller samples or by producing larger samples.

In the study, the acoustic analysis defined the boundaries of the cavity as ‘acoustically rigid walls’. Although the calculated modes closely matched the measured sound absorption peaks, further theoretical and numerical research is needed. This research must consider complex geometrical and vibro-acoustic interactions, as well as material anisotropy, to develop a more comprehensive acoustical model. Another approach would be to study the mechanical system using an electro-acoustical analogy.

## Figures and Tables

**Table 1:** Parameter values of the tested origami structures.

Sample	$l_{x}$ [mm]	$l_{y}$ [mm]	$l_{z}$ [mm]	θ [°]	*m* [/]	*n* [/]	$\tau_{g}$ [/]	*A* [m^2^]
A	72	/	45	180	11.04	/	1.00	1.0
B	72	72	45	113	11.02	10.96	1.18	1.2
C	72	72	45	55	11.05	10.99	2.17	1.7

## Data Availability

All data is presented in the paper.

## Abbreviations

The following abbreviations are used in this manuscript:

LDV	Laser Doppler Vibrometer
FEM	Finite Element Method
